# A Scanning Electron Microscopic Comparison of the Cleaning Efficacy of Endodontic Irrigants

**Published:** 2007-10-02

**Authors:** Seddigheh khedmat, Afshin Shadi

**Affiliations:** 1 *Department of Endodontics, Dental School and Dental Research Center, Tehran University of Medical Sciences, Tehran, Iran*; 2 *General Practitioner, Tehran, Iran*

**Keywords:** EDTA, Irrigating Solution, Smear Clear, Sodium Hypochlorite, SEM

## Abstract

**INTRODUCTION:** The aim of this study was to compare the cleaning efficacy of three irrigants used during and after instrumentation.

**MATERIALS AND METHODS:** Eighty-four single rooted human teeth were randomly divided into 7 groups, 12 cases each, and canals were instrumented with Mtwo rotary systems. 5.25% sodium hypochlorite, Smear Clear and 17% EDTA were used for irrigation of the canal during and/ or after instrumentation. After completion of instrumentation, all canals were dried with paper points and prepared to examine by scanning electron microscope. All SEM photomicrographs were scored at the coronal, middle and apical thirds of canals in each group. The data were statistically analyzed using Mann-Whitney and Kruskal-Wallis tests.

**RESULTS:** In the group irrigated with Smear Clear alone, the coronal thirds of the canals were significantly cleaner than middle thirds (P=0.013) and apical thirds (P=0.028). There were less smear layer in the coronal thirds compared to apical thirds (P=0.047) in the group irrigated with Smear Clear and NaOCl alternately .There were significantly more smear layer in the apical thirds compared to coronal thirds (P<0.001) and middle thirds (P=0.007) in the group that Smear Clear and NaOCl were used as final irrigations. There was not any significant difference between three-thirds of the canals in other groups. Comparison of the all groups showed a statistically significant difference (P<0.001) in the amount of debris and smear layer remaining at all three levels of the canals**.**

**CONCLUSION:** The findings of this study suggested that a final rinsing by combination of a chelating agent like EDTA or Smear Clear and NaOCl is necessary to obtain favorable clean wall of root canals.

## INTRODUCTION

It is well known that the irrigation of the root canal plays a critical role in determining the success of endodontic therapy (1).

Goldman *et al.* demonstrated that in canals prepared without irrigating solutions, the quantity of debris found after instrumentation is higher than the cases with irrigants use (2). Debris is defined as dentin chips, and residual vital or necrotic pulp tissue attached to the root canal wall, which is mostly infected (3). On the other hand, dentin instrumentation causes the formation of a smear layer that covers the whole surface of the root canal. the smear layer consist of a superficial layer on the surface of the canal walls with approximately 1 to 2 µm of thickness and a deeper layer packed into the dentinal tubules up to 40 µm (4). This layer contains necrotic tissue and bacterial remnants and its presence might prevent the penetration of intra canal medicaments into the dentinal tubules and negatively affects the adaptation of the filling materials; thus, removal of smear layer is assumed to be beneficial. Therefore, not only the removal of pulpal remnants is necessary, but also smear layer removal from root canal walls is equally important (5,6). Koskinen *et al.* studied the cleaning ability of various chemical substances using scanning electron microscopy and found that none of the substances acted on the organic residues and the mineralized portion, simultaneously; thus, each could be associated with or completed by the other (7). Sodium hypochlorite is the most used root canal irrigant. Despite good antibacterial and dissolving effects on the necrotic tissue, this solution is not able to remove the smear layer thoroughly because it is directly effective on organic debris (7).

**Table 1:** Experimental groups

**Group**	**During instrumentation**	**After instrumentation**
A (Control+)	Distilled water	___
B	5.25% NaOCl	___
C	Smear Clear	___
D	Smear Clear and 5.25% NaOCl	___
E	Smear Clear and 5.25% NaOCl	1 mL Smear Clear for 1 min then 3 mL 5.25% NaOCl
F	5.25% NaOCl	1 mL Smear Clear for 1 min then 3 mL 5.25% NaOCl
G (Control-)	5.25% NaOCl	1 mL 17% EDTA for 1 min then 3 mL 5.25% NaOCl

To obtain a complete removal of the smear layer, both organic and inorganic components, combination of sodium hypochlorite and a chelating agent like ethylene diamine tetra acetic acid (EDTA) is recommended (5,8,9). It has been reported that 17% EDTA followed by 5% sodium hypochlorite remove the smear layer in 1min if the fluid is able to reach the root canal wall surface (10).

Several studies have shown that EDTA application more than 1min and in volume more than 1 mL caused dentinal erosion (9-11). An efficient removal of the smear layer is accomplished with a final rinse of 1 mL of 17% EDTA for 1 min followed by 3 mL of 5.25% sodium hypochlorite (9).

Smear Clear (Sybron Endo, CA) is a recently introduced product for removal of smear layer. It is a 17% EDTA solution with 2 additional proprietary surfactants. It may be speculated that irrigation with Smear Clear would be more efficient compared to EDTA. The only published study to date by Lui *et al. *demonstrated that the surfactants in Smear Clear did not improve its efficacy in smear layer removal compared to EDTA alone (12).

The aim of this *in vitro* study was to compare the efficacy of three endodontic irrigants (5.25% sodium hypochlorite, Smear Clear and 17% EDTA) and their combination in removal of pulpal debris and smear layer.

## MATERIALS AND METHODS

Eighty-four extracted single-rooted human teeth with straight roots were selected. The crown of each tooth was removed at the level of the cemento-enamel junction to ensure good visibility of the canal, optimal access and obtain root segments of approximately 12mm in length. After preparing an access for each tooth, a K-type file (#15) was used to determine the working length by penetrating the apical foramen and pulling back to the clinically visible apical foramen. The working length was calculated 1mm short of this position. Teeth with apical diameters larger than #15 were excluded from the study. Teeth with sclerotic canals or with an altered apex were not included. The specimens were prepared with rotary Mtwo instruments (VDW, Munich, Germany). Five instruments were used at the working length in each canal according to the manufacturer's instruction with following sequence: a) #10, 0.04 taper, b) #15, 0.05 taper, c) #20, 0.06 taper, d) #25, 0.06 taper e) # 30, 0.05 taper. Each instrument was used only in five canals. Between the use of instruments, canals were irrigated with 2 mL of the experimental irrigating solutions. The solutions were introduced into the canals by using a 30 gauge needle to within nearly 1mm of the working length. Eighty-four selected teeth randomly divided into 7 groups of 12 teeth. The types of irrigants used during and after instrumentation in different groups are shown in Table 1.

After completion of Instrumentation, all canals were dried with paper points then two longitudinal grooves were prepared on the palatal/lingual and buccal surfaces of each root with a diamond disc to facilitate vertical splitting of the specimens into two halves with a chisel. The section with most visible part of the apex was conserved and coded. The coded, halved specimens were then mounted on metallic stub, gold-sputtered and examined by a SEM (DSM 940A Zeiss, Hamburg, Germany).

**Figure 1:**
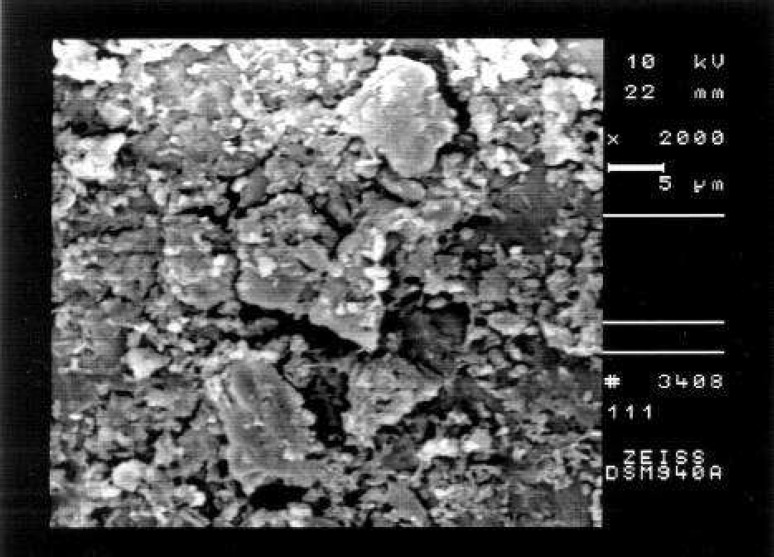
An example of the prepared surface of coronal third of a canal in Group A showing total coverage by debris and smear layer (Score 3, ×2000 Mag.)

After general survey of the canal wall from the apex to the most coronal part, four SEM photomicrographs were obtained at ×1000 and ×2000 magnifications at each coronal, middle and apical third. 12 SEM photomicrographs were taken of the canal walls at the 2, 6 and 10-mm from the apical foramen of each specimen. Some areas of dentin were also observed at ×5000 or ×200 magnifications. The amounts of debris and smear layer at the coronal, middle and apical portions of each canal scored according to the following criteria (12).

**A:** Smear score:

**0**) No smear layer. All dentinal tubules were clean and open, **1**) Some dentinal tubules were open with smear layer covering some of the openings of the dentinal tubules, and **2**) All dentinal tubules are covered by smear layer

**B:** Debris score:

**0**) No debris was present, **1**) Few debris particles were present, and** 2**) Large amounts of debris particles were present.

**Table 2:** Debris scores distribution at three-thirds of root canals in each group (%)

	**Coronal**			**Middle**			**Apical**		
	**0**	**1**	**2**	**0**	**1**	**2**	**0**	**1**	**2**
**A**	0	9.1	90.9	0	0	100	0	0	100
**B**	100	0	0	80	20	0	60	40	0
**C**	63.6	36.4	0	3.1	72.7	18.2	18.2	54.5	27.3
**D**	100	0	0	90.9	9.1	0	63.6	36.4	0
**E**	90.9	9.1	0	90.9	9.1	0	81.9	18.2	0
**F**	100	0	0	91.7	8.3	0	83.3	16.7	0
**G**	100	0	0	91.7	8.3	0	75	25	0

**Figure 2:**
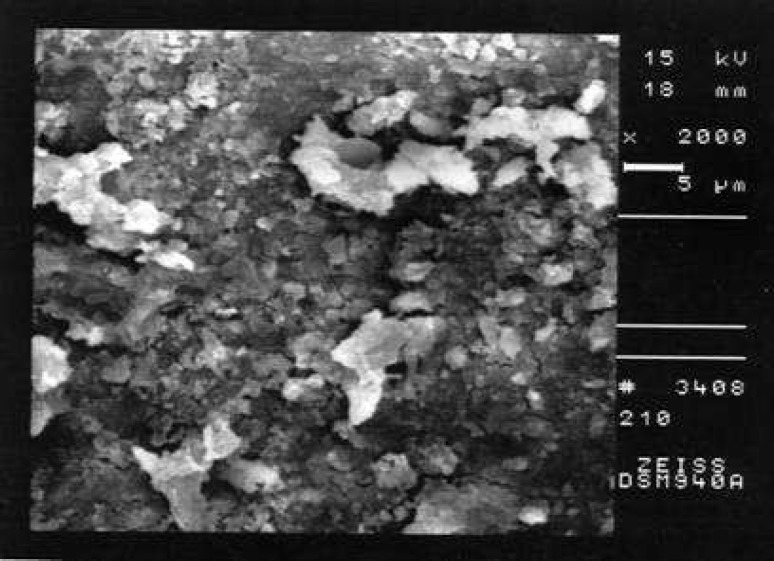
An example of the prepared surface of the coronal third of a canal in Group B (Smear score 3, debris score 1, ×2000 Mag.)

All SEM photomicrographs were scored by an endodontist who was unaware of coding system to exclude observer bias. Evaluation was repeated twice for the first 20 specimens to ensure intra examiner consistency.

Data were analyzed with the Kruskal-Wallis and Mann-Whitney U tests. P-Values were computed and compared with the P=0.05 level.

## RESULTS

Debris and smear scores distribution at the coronal, middle and apical thirds of root canals in each group are presented in Table 2 and Table 3. Three thirds of the canals in each group were compared with respect to the debris and smear layer.

Descending order of debris/smear removal was observed from coronal to apical areas. There was not any significant difference in debris and smear scores between three-thirds of the canals in groups A, B, F, and G.

**Table 3:** Smear scores distribution at three-thirds of root canals in each group (%)

	**Coronal**			**Middle**			**Apical**		
	**0**	**1**	**2**	**0**	**1**	**2**	**0**	**1**	**2**
**A**	0	0	100	0	0	100	0	0	100
**B**	0	0	100	0	0	100	0	0	100
**C**	9.1	54.5	36.4	0	54.5	45.5	0	9.1	90.9
**D**	9.1	90.9	0	9.1	81.9	9.1	0	54.5	45.5
**E**	81.8	18.2	0	63.6	36.4	0	0	90.9	9.1
**F**	33.3	66.7	0	33.3	66.7	0	25	50	25
**G**	66.7	33.3	0	66.7	25	8.3	41.7	58.3	0

**Figure 3:**
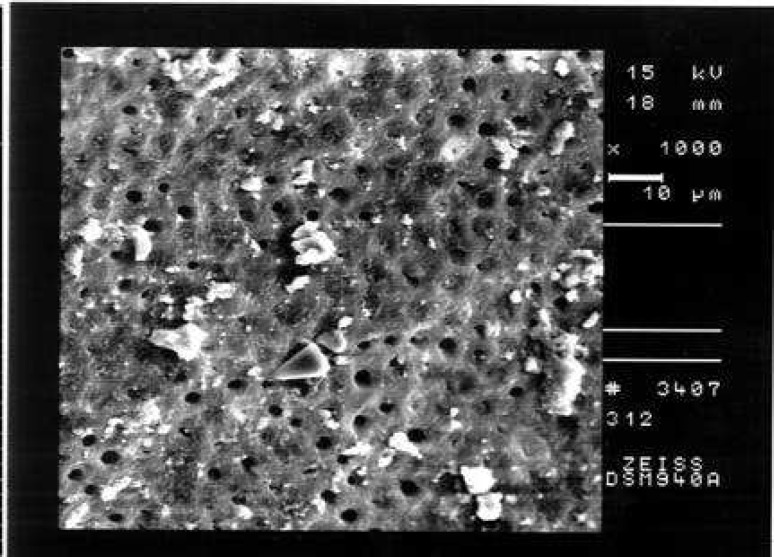
An example of the prepared surface of the middle third of a canal from Group C (smear score 2, debris score 2, ×1000 Mag.)

**Figure 4:**
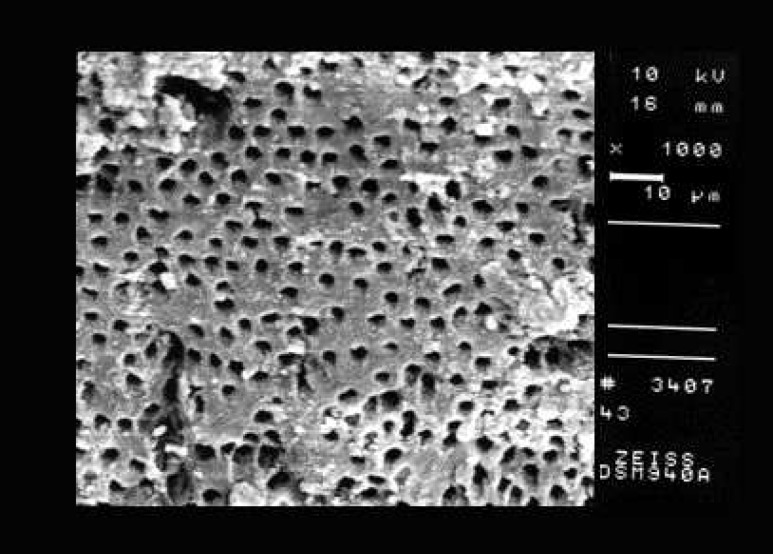
An example of the prepared surface of apical third of a canal from Group D (smear score 2, debris score 1, ×1000 Mag.)

**Figure 5:**
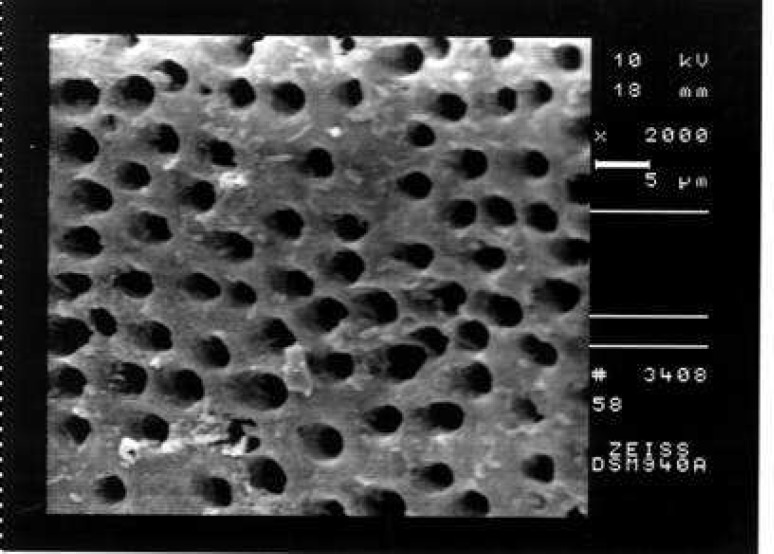
An example of the prepared surface of middle third of a canal from Group E (smear score 1, debris score 1, ×2000 Mag.)

The groups F and G have the same results in group C, the coronal thirds of the canals had significantly less smear layer and debris compared to middle thirds (P=0.013) and apical thirds (P=0.028); In group D, there were less smear layer in the coronal thirds compared to apical thirds (P=0.047), and there was significantly more smear layer in the apical thirds of group E than coronal thirds (P<0.001) and middle thirds (P=0.007).

**Figure 6:**
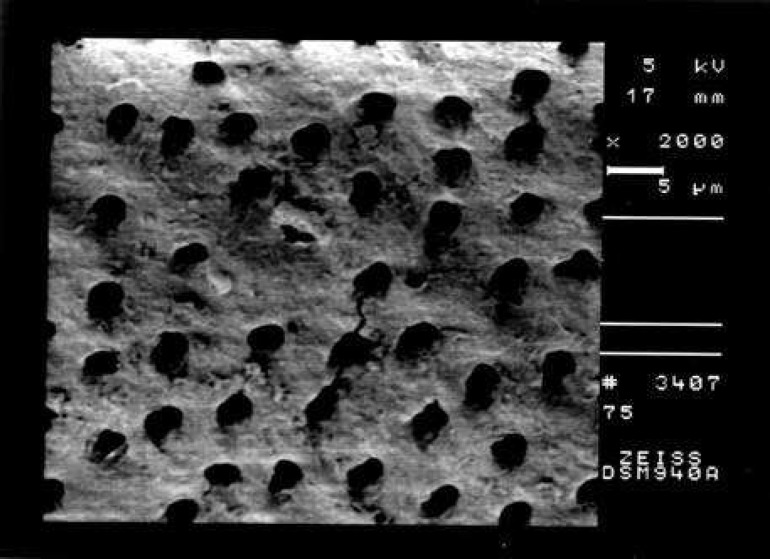
An example of the prepared surface of apical third of a canal from Group G (smear score 1, debris score 1, ×2000 Mag.)

Comparison of the seven groups showed a statistically significant difference in the amount of remaining debris and smear layer at all three levels of canals (P<0.001) (Figure 1),(Figure 2),(Figure 3),(Figure 4),(Figure 5),(Figure 6).

Removal of the smear layer was significantly different between group A and all other groups except for group B.

Samples in group C, except for apical thirds, were significantly cleaner than those irrigated with distilled water and 5.25% sodium hypochlorite; this group and group F was not significantly different from group D.

All three- thirds of the canals in D, E, F and G groups were significantly cleaner of smear layer than groups A and B; the difference was not significant in debris removal between these four groups and group B, but significant with group C (p<0.03). No significant difference was observed among these four groups.

Samples in group E and G were cleaner of smear layer than those of group C (p=0.001, p<0.005 respectively); in group F canals were significantly cleaner at the middle (p=0.009) and apical thirds (p=0.004) than group C.

Except for the apical thirds, root canals in group E were cleaner of smear layer at the coronal (p=0.002) and middle thirds (p=0.019) than those in group D.

There were significant differences in smear layer removal between group D and G at the coronal (p=0.019), middle (p=0.032) and apical (p=0.004) thirds of the canals.

Debris removal in root canals irrigated with NaOCl was more at all levels compared to those irrigated with distilled water.

Root canals irrigated with NaOCl were significantly cleaner of debris at the middle (p=0.003) and apical (p=0.043) thirds than those irrigated with Smear Clear.

There was no significant difference between groups E, F and G with respect to smear layer and debris removal.

## DISCUSSION

In this study, a scanning electron microscope was used to assess the effectiveness of various irrigants and final rinse to remove the debris and smear layer. Analysis of the dentinal walls of all the specimens in the experimental groups demonstrated that cleaning have been more effective on the coronal and middle thirds than on the apical third. It is possible that the size of the canals in these thirds, allowed better circulation and action of the irrigating solution, making the complete removal of the smear layer and debris more possible. These results are in agreement with those of various authors who have observed an effective cleaning action on these thirds even when different volumes of solutions and times of irrigation were employed (11,13-15).

The results showed no significant difference in the ability of distilled water and 5.25% sodium hypochlorite to remove the smear layer from the surfaces of instrumented root canals, because both irrigants were ineffective. These findings are in agreement with those observed in previous investigations (8,11,16).

Comparing the results of group C (Smear Clear alone as an irrigant) with group E and F (Smear Clear and various volume of NaOCl as an irrigant during and after instrumentation) indicates that using of sodium hypochlorite to assist Smear Clear significantly improves its cleaning ability because sodium hypochlorite as an irrigant is able to dissolve debris and necrotic tissue and to remove the organic part of the smear layer. According to Abou-Rass and Patonai reduction of surface tension property of endodontic solutions improves their flow into narrow root canals (17).

Therefore, it maybe speculated that addition of two surfactants should improve penetration of 17% EDTA in the root canal. However our study showed that the surfactants in Smear Clear (group E and F) did not improve its performance in canal cleaning compared to EDTA alone (group G). This result confirmed the finding of Lui *et al.* about smear clear and EDTA (12).

The study of Scelza *et al.* showed that the addition of tergentor as a surfactant to EDTA (EDTA-T) did not allow the chelation of Ca^++^ with the same intensity as EDTA alone and EDTA-T had fewer efficacies in terms of Ca^++^ removal in comparison with 17% EDTA (18). Recently, it was shown that reducing the surface tension of endodontic chelator solutions did not improve their calcium chelating ability and that the addition of a wetting agent to a chelator solution is unnecessary (19).

In vivo, root canals are usually wet, and the surface tension of endodontic solutions may not play role in their penetration ability (20).

The best results in this study were obtained when a final irrigation were used (E to G groups), therefore using of a final flush of Smear Clear/EDTA followed by NaOCl is suggested.

## CONCLUSION

Based on the results of this *in vitro* study, using of the irrigants during and after instrumentation is necessary to obtain favorable clean root canal system.

## References

[1] Torabinejad M, Walton R (2002). Principles and practice of endodontics.

[2] Goldman M, Goldman LB, Kronman JH, Lin PS (1981). The efficacy of several irrigating solutions for endodontics: a scanning electron microscopic study. Oral Surg Oral Med Oral Pathol.

[3] Hulsmann M, Rummelin C, Schafers F (1997). Root canal cleanliness after preparation with different endodontics hand pieces and hand instruments: a comparative SEM investigation. J Endod.

[4] Mader CL, Baumgartner JC, Peters DD (1984). Scanning electron microscopic investigation of the smeared layer on root canal walls. J Endod.

[5] Yamada RS, Armas A, Goldman M, Lin PS (1983). A Scanning electron microscopic comparison of a high volume flush with several irrigating solutions Part 3. J Endod.

[6] Scelza MFZ, Antoniazzi JH, Scelza P (2000). Efficacy final irrigation- A scanning Electron microscopic evaluation. J Endod.

[7] Koskinen KP, Meurman JH, Stenvall H (1980). Appearance of chemically treated root canal walls in the scanning electron microscope. Scand J Dent Res.

[8] Grandini S, Balleri P, Ferrari M (2002). Evaluation of Glyde file pre in combination with sodium hypochlorite as a root canal irrigant. J Endod.

[9] Crumpton BJ, Goodell GG, Mc Clanahan SB (2005). Effects on smear layer and debris removal with varying volumes of 17% REDTA after rotary Instrumentation. J Endod.

[10] Calt S, Serper A (2002). Time-dependent effects of EDTA on dentin structures. J Endod.

[11] Torabinejad M, Khademi A, Babagoli J, Cho Y, Johson WB, Bozhilov K, Kim J, Shabahang S (2003). A new solution for the removal of the smear layer. J Endod.

[12] Lui JN, Kuab HG, Chen NN (2007). Effect of EDTA with and without surfactants or ultrasonics on removal of smear layer. J Endod.

[13] Baumgartner JC, Mader CL (1987). A scanning electron microscopic evaluation of four root canal irrigant regimens. J Endod.

[14] Abbott PV, Heijkoop PS, Cardacis C, Hume WR, Heithersay GS (1991). An SEM study of the effects of different irrigation sequences and ultrasonics. Int Endod J.

[15] Teixeira CS, Felippe MCS, Felipp WT (2005). The effect of application time of EDTA and Naocl on intra canal smear layer removal: an SEM analysis. Int Endod J.

[16] Torabinejad M, Cho Y, Khademi AA, Bakland LK, Shabahang SH (2003). The effect of various concentration of sodium Hypochlorite on the ability of MTAD to remove the smear layer. J Endod.

[17] Abou-Rass M, Patonai FJ (1982). The effects of decreasing surface tension on the flow of irrigating solution in narrow root canals. Oral Surg Oral Med Oral Pathol.

[18] Scelza MFZ, Teixeira AM, Scelza P (2003). Decalcifying effect of EDTA-T, 10% citric acid and 17% EDTA on root canal dentin. Oral Surg Oral Med Oral Pathol Oral Radiol Endod.

[19] Zehander M, Schicht D, Sener B, Schmidlin P (2005). Reducing surface tension in endodontic chelator solutions has no effect on their ability to remove calcium from instrumented root canals. J Endod.

[20] Zehnder M (2006). Root canal irrigants. J Endod.

